# Tunable interplay between exchange coupling and uniaxial magnetic anisotropy in epitaxial CoO/Au/Fe trilayers

**DOI:** 10.1038/s41598-023-38098-6

**Published:** 2023-07-05

**Authors:** H. Nayyef, E. Świerkosz, W. Janus, A. Klimeczek, M. Szpytma, M. Zając, P. Dróżdż, A. Kozioł-Rachwał, T. Ślęzak, M. Ślęzak

**Affiliations:** 1 Faculty of Physics and Applied Computer Science, AGH University of Krakow, Kraków, Poland; 2 National Synchrotron Radiation Centre SOLARIS, Jagiellonian University, Kraków, Poland; 3 Institute of Physics, Maria Curie-Sklodowska University, Lublin, Poland

**Keywords:** Applied physics, Condensed-matter physics, Nanoscience and technology, Materials science, Magnetic properties and materials

## Abstract

We show that the interaction between ferromagnetic Fe(110) and antiferromagnetic CoO(111) sublayers can be mediated and precisely tuned by a nonmagnetic Au spacer. Our results prove that the thickness of the Fe and Au layers can be chosen to modify the effective anisotropy of the Fe layer and the strength of the exchange bias interaction between Fe and CoO sublayers. Well-defined and tailorable magnetic anisotropy of the ferromagnet above Néel temperature of the antiferromagnet is a determining factor that governs exchange bias and interfacial CoO spins orientation at low temperatures. In particular, depending on the room temperature magnetic state of Fe, the low-temperature exchange bias in a zero-field cooled system can be turned “off” or “on”. The other way around, we show that exchange bias can be the dominating magnetic anisotropy source for the ferromagnet and it is feasible to induce a 90-degree rotation of the easy axis as compared to the initial, exchange bias-free easy axis orientation.

## Introduction

Magnetic anisotropy (MA) originates from the coupling between the electron spin and the orbital moment and is a key parameter for the magnetic materials properties. MA is fundamentally important and crucial for nanoscale applications as it both determines the preferential orientation of magnetic moments and is required for long range magnetic order in thin films and nanostructures^1,2^. In case of ferromagnets (FM) the magnetic anisotropy originates either from their shape^3^, crystalline anisotropy^4,5^, or in case of antiferromagnet/ferromagnet^6,7^ systems from the interactions with the neighboring antiferromagnet (AFM). In the last AFM/FM case the well-known exchange bias (EB) effect may occur due to the interfacial exchange coupling between FM and AFM layers^8–10^. The exchange bias, manifested usually by the horizontal displacement and increased coercivity of the magnetic hysteresis loop, is associated with the induced unidirectional anisotropy in the ferromagnetic layer. Although exchange bias has been intensively studied in past^11–13^ and more recently^14–18^ and has been also applied in magnetic sensors, memory devices^19^ and nowadays even in spin orbit torque (SOT) prototypes^20^, still many controversial issues remain concerning its microscopic mechanism^21–24^. Usually the easiest experimentally accessible quantity is the exchange bias induced horizontal shift of the magnetic hysteresis loop. Unfortunately, concluding from exchange bias shift field alone is often difficult or even impossible as both ferromagnet and antiferromagnet MAs can play decisive role for establishing and magnitude of EB. For instance, too low MA of AFM may result in the so-called rotatable AFM spins that follow the FM magnetization during reversal and thus give rise to enhanced coercivity only^25,26^ while the magnetic hysteresis loop remains fully symmetric with respect to its zero-field axis. Providing the intrinsic MA of AFM is sufficiently large in order to turn on the EB, it can happen that the induced unidirectional MA is noncollinear with the orientation of external magnetic field used during growth of the film or field cooling (FC) procedure^27^. The situation is further complicated by the fact that also MA of FM layer can lead to the significant EB in some cases when it is not expected, for example when magnetic field during magnetization reversal is perpendicular to the field applied during FC procedure ^28–30^. All these facts call for the study of EB single system in which both MAs and strength of AFM-FM interaction are well defined and fully controllable. The aim of the present report is to study the interplay between uniaxial and unidirectional magnetic anisotropies in FM/AFM exchange coupled system and to tune it either by controllable indirect exchange coupling strength or by adjustable balance of surface and volume contributions to magnetic anisotropy in FM layer. As a result we expect possibility to reorient the easy axis of either of the two (uniaxial and unidirectional) MAs. The target of our research were epitaxial CoO(111)/Au(111)/Fe(110) trilayers grown on W(110) single crystal. Using wedged ferromagnetic bottom layer of the stack allowed us to control its uniaxial in-plane magnetic anisotropy including both smooth evolution of its strength and the change of its sign. The latter is manifested by the well-known spin reorientation transition (SRT)^31–33^, in which at the critical Fe thickness, 90-degree, in-plane switching of the Fe easy axis is observed^34–38^. The FM-AFM interaction strength and resulting unidirectional MA were controlled by the non-magnetic (NM) spacer thickness in the wedged Au sublayer. To trace the evolution of the effective in-plane MA of the FM layer, unidirectional MA axis and the EB effect strength, the magnetic hysteresis loops were measured and analyzed in a two-dimensional (d_Fe_, d_Au_) space.

## Experimental details

**Figure 1:**
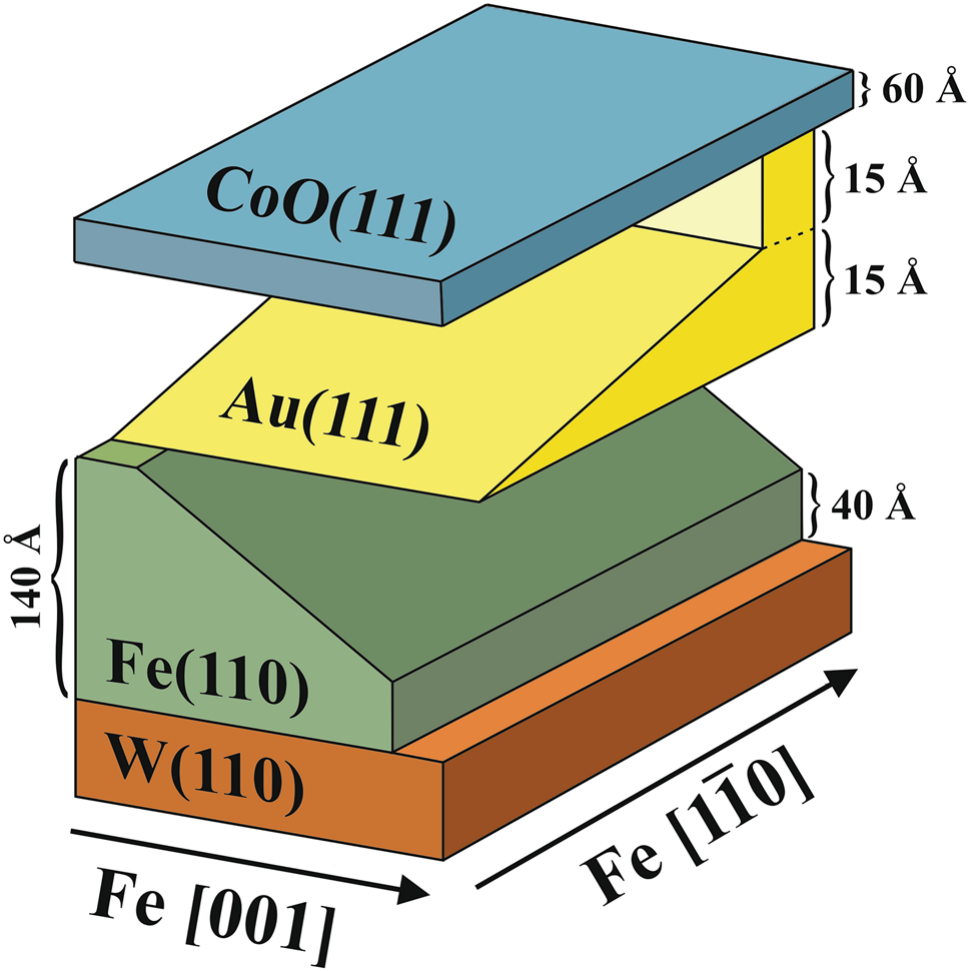
Schematic sketch of the double wedged CoO(111)/Au(111)/Fe(110) sample.

Epitaxial CoO(111)/Au(111)/Fe(110) trilayers were in situ deposited on W(110) surface using molecular beam epitaxy. Firstly, wedged Fe layer with the thickness range 40 – 140 Å was grown on clean W(110) single crystal at room temperature and post-annealed at 675 K to obtain atomically smooth (110) surface. Next, a 0 -15 Å wedge of Au spacer oriented orthogonally to Fe wedge was deposited and also post-annealed at 500 K in order to obtain high quality Au(111)/Fe(110) bilayer, see schematic drawing of the sample in Fig. 1 where also the additional 30 Å thick Au sample area called ‘Au chimney’ is shown. The ‘chimney’ area was prepared to provide region where AFM-FM interaction is fully suppressed. The whole sample area was covered by homogenous 60 Å thick CoO(111)^39,40^ layer prepared at room temperature by reactive deposition of metallic Co at partial pressure of molecular oxygen 1 × 10^−6^ mbar. At each step of the preparation procedure low energy electron diffraction (LEED) patterns were collected confirming the high quality crystalographic structure of subsequent epitaxial sublayers, please see Supplementary Material.

## Results and discussion

**Figure 2:**
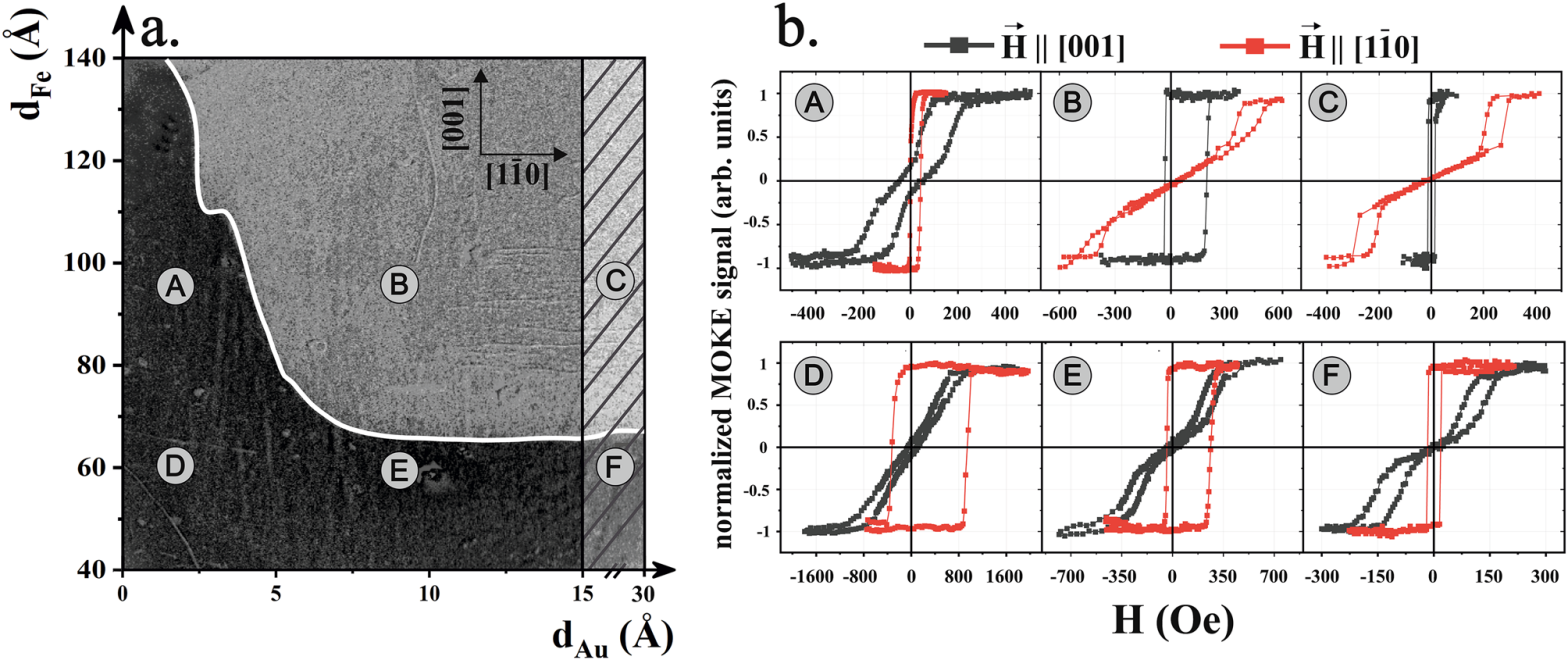
(**a**) Differential in situ MOKE microscope image of the sample surface at the remanent state. The magnification of the MOKE microscope was intentionally tuned to cover the chosen field of view which is 4.6 mm (horizontal) × 4.0 mm (vertical). (**b**) Selected area (A, B, C, D, E and F) MOKE hysteresis loops for two orthogonal directions of the external magnetic field applied during magnetization reversal. C and F sample areas correspond to fixed 30 Å thickness of Au (‘chimney’). The magnetic hysteresis loops were collected ex situ using laser with its spot on the sample roughly ~ 0.25 mm wide that corresponds to the averaging of magnetic properties over a finite thickness intervals ∆d_Fe_ =  ~ 5 Å and ∆d_Au_ =  ~ 0.8 Å for the Fe and Au wedges, respectively. Presented MOKE results were obtained at 80 K.

The magnetic properties of the CoO(111)/Au(111)/Fe(110) trilayers were firstly in situ imaged using the longitudinal magneto-optic Kerr effect (MOKE) microscope. Figure 2a shows a differential MOKE image of a selected area of the sample in its remanent state after application of external magnetic field oriented along Fe[1$\overline{1 }$0] in-plane direction. To enhance the magnetic contrast and to highlight characteristic features present in the sample, we subtracted a reference image taken at a small external magnetic field along [1$\overline{1 }$0] from the image taken at remanence (H = 0). Consequently, the dark area is where the remanence magnetization remained along the saturation direction, [1$\overline{1 }$0], whereas the brighter area corresponds to the [001] magnetization direction in the remanent state, as concluded for example from comparison of hysteresis curves presented for regions of interest E and B or F and C. Due to relatively poor quality of hysteresis loops acquired by in situ MOKE microscope, the results presented in Fig. 2b were obtained in ex situ standard MOKE setup (not microscopic mode). As we checked with X-ray magnetic linear and circular dichroism (XMLD and XMCD, respectively) techniques, CoO overlayers efficiently protect the samples from ambient conditions and ensure that their magnetic properties can be followed ex situ without any additional capping layers.

Six representative sample regions of interests (ROIs: A, B, C, D, E and F) are marked in Fig. 2a and corresponding MOKE hysteresis loops are presented in Fig. 2b. For both D and A ROIs typical square (however exchange biased) hysteresis loops are observed for **H ||** [1$\overline{1 }$0] (red plots in Fig. 2b) which means that for this particular Au thickness (~ 2Å) [1$\overline{1 }$0] is the easy axis of Fe independently on its thickness, at least up to d_Fe_ = 140 Å. (Please note, that for d_Au_ < 2Å the SRT to Fe[001] orientation of magnetization would take place above d_Fe_ = 140 Å which is the highest studied Fe thickness in this report.) This is clearly confirmed by the magnetic hysteresis loops measured in **H ||** [001] complementary MOKE geometry where characteristic hard axis loops with close to zero magnetization in remanent state are observed (grey plots in Fig. 2 b). Different conclusion can be withdrawn from comparison of ROIs E and B (d_Au_ = 9 Å) and also F and C (d_Au_ = 30 Å, ‘chimney’). In both these cases, with increasing Fe thickness the in-plane SRT from Fe[1$\overline{1 }$0] to Fe[001] bulk-like easy axis of Fe is seen. Specifically, with increasing Fe thickness magnetic hysteresis loops change from ‘square’ to ‘hard-like’ for **H ||** [1$\overline{1 }$0] geometry (red color) and vice versa in case of **H ||** [001] (grey color). Additionally, for the ‘chimney’ area the EB effect is fully suppressed and magnetic hysteresis loops both before (F) and after (C) SRT are symmetric with respect to zero-field axis. All these preliminary observations allow us to treat the image in Fig. 2a as an overview of the sample magnetic properties in the two-dimensional (d_Fe_, d_Au_) space. The continuous white line separating the dark and bright sample areas in Fig. 2a marks the critical SRT border in this space. Relatively drastic decrease of the critical SRT thickness with d_Au_ and hints of its oscillations (mainly visible around d_Au_ = 2.5 Å) stay in good agreement with our previous report on quantum well states in uncovered Au(111)/Fe(110) bilayers^41^. The observed difference (i.e. lack of clear periodic oscillations with increasing d_Au_) results from strong interaction of Fe with AFM CoO for low spacer thicknesses. Already from the data presented in Fig. 2b some quantitative conclusions on EB and unidirectional MA can be pointed out. For example, please note that EB effect follows the thickness induced SRT in Fe. Specifically, for given d_Au_ and with increasing Fe thickness (for example SRT_E→B_) the strongly exchange biased hysteresis loop (E, red in Fig. 2b) switches to ‘hard-like’ and symmetric (H_EB_ = 0) one (B, red) while the corresponding **H ||** [001] loop (B, grey) becomes shifted along external magnetic field axis. This means that SRT in Fe resulting from the change of its uniaxial MA easy axis drives also the in-plane rotation of the unidirectional MA axis in the system. The same effect can be also noticed for example for d_Au_ driven SRT_A→B_.

**Figure 3:**
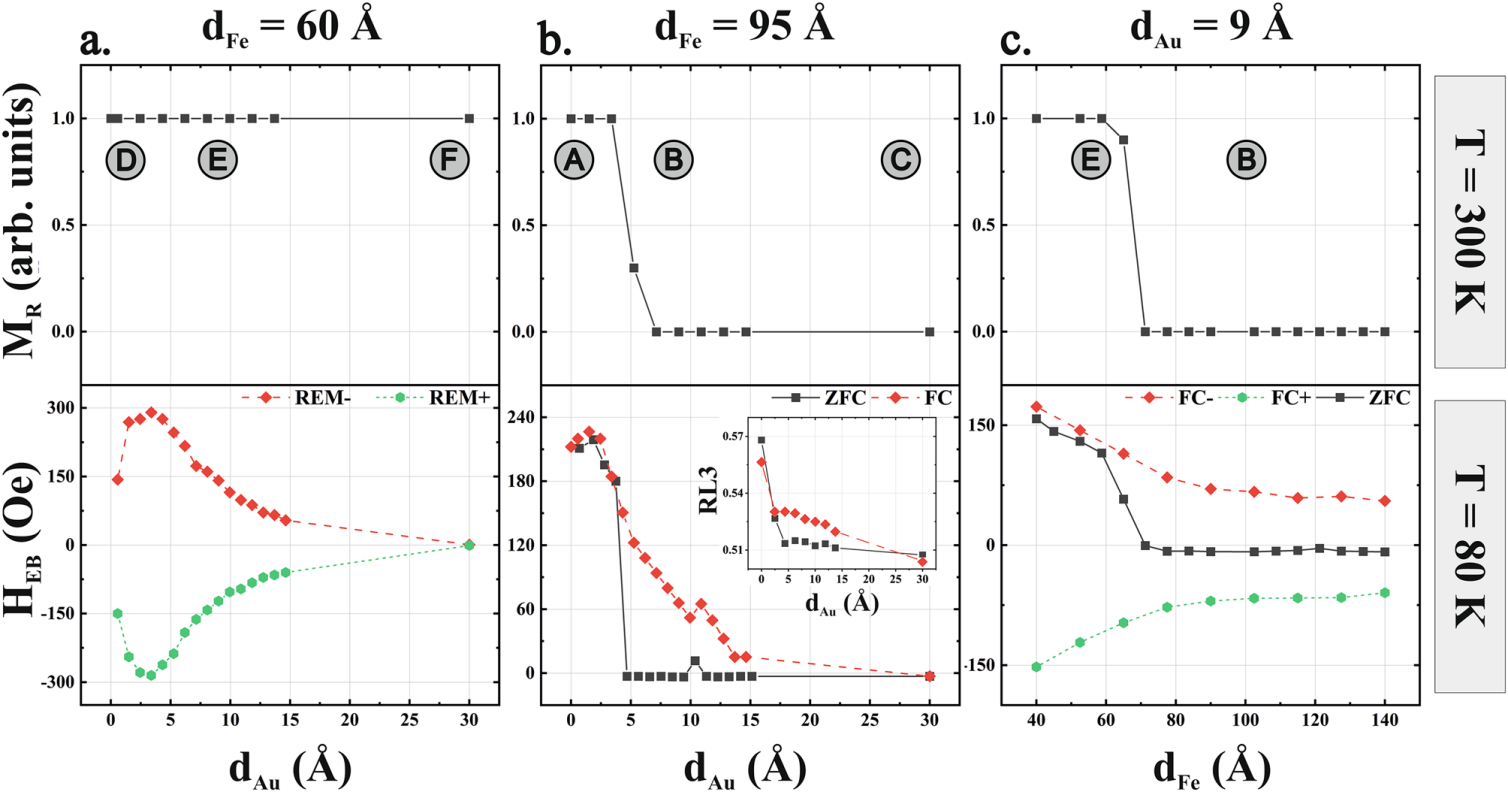
(Top panel) Normalized Fe magnetization in remanence (M_R_) state as determined from MOKE hysteresis loops measured in **H ||** [1 $\overline{1 }$ 0] MOKE geometry, at 300 K. (bottom panel) Corresponding 80 K exchange bias field vs d_Au_ (**a** and **d**) and vs d_Fe_ (**c**) for three analyzed scenarios, see text for details. Inset in bottom (**b**) shows the 80 K XMLD (R_L3_) dependence on Au thickness for both zero-field (ZFC) and FC cooled sample. Letters in top panel plots schematically mark the selected ROIs defined previously in Fig. 2 sample map.

In order to provide deeper insight into EB and unidirectional MA dependence on the uniaxial MA of Fe the magnetic hysteresis loops were systematically acquired in **H ||** [1$\overline{1 }$0] geometry as a function of d_Au_ (sequences D → E → F and A → B → C) and d_Fe_ (exemplary E → B sequence), please see results in Fig. 3a,b,c, respectively. The top row in Fig. 3 contains plots of normalized Fe magnetization in remanence state at room temperature, so above CoO Néel temperature (T_N_ ~ 293 K). Three interesting scenarios are chosen for analysis i.e. the SRT-free situation (Fig. 3a) and both d_Au_ and d_Fe_ induced SRTs (Fig. 3 b and c).

In first of these cases (no SRT) the sample was first cooled down in its positive remanence state (after saturating the sample in positive **H**) and H_EB_(d_Au_) dependence was followed at 80 K, please see green plot at the bottom of Fig. 3a, marked as ‘REM + ’. Clearly, continuous although non-monotonous H_EB_ dependence on the Au thickness is observed with its maximum magnitude ~ 300 Oe for ~ 1 monolayer thick Au spacer, smooth decay to ~ 70 Oe at d_Au_ = 15Å and fully suppressed EB at the ‘chimney’ d_Au_ = 30 Å sample region. Interestingly, the insertion of a single Au monolayer enhances the EB effect in CoO/Au/Fe trilayer by ~ 100% as compared to direct coupling in CoO/Fe bilayer (d_Au_ = 0). Similar effect has been reported for polycrystalline IrMn/NM/CoFe systems with NM = {Ag, Al, Ti} and intentionally very rough AFM/FM interface prepared^42^. Enhancement of EB upon insertion of thin non-magnetic spacer was also observed in Refs.^43–45^ and ascribed to partial reduction of magnetic frustration at the AFM/FM interface^43,45^ or to increased net FM component along biasing field^44^. In the present report the quality of the LEED diffraction images (please see Supplementary Material) observed on CoO surface is significantly worse as compared to sharp diffraction spots of Fe(110) sublayer which slightly mimics the rough interface scenario described in Ref.^42^ However, in the present report one can also note almost exact coincidence between the observed maximum H_EB_ in Fig. 3a and clear local maximum of the critical SRT border line in Fig. 2a for d_Au_ = 2.5 Å. The latter could be ascribed to the quantum well states (QWS) recently reported for Au(111)/Fe(110) uncovered bilayers^41^. Such QWS can not be ruled out as a driving force for the observed enhanced, Au-mediated AFM/FM exchange interaction, however the absence of subsequent oscillations of H_EB_ for thicker Au does not support such interpretation. On the other hand, interfacial oxidation of Fe surface during the CoO growth that has a negative impact on exchange bias effect is suppressed by the presence of ultrathin Au spacer. In parallel the exchange interaction across such thin Au film is still strong enough to ensure significant EB effect. The Au film becomes efficient barrier against oxidation process after completion of its first atomic layer, as confirmed by XAS spectra of Fe shown in Supplementary Material, Fig. 6S. After cooling down the sample in its second, ‘negative’ remanence state denoted in Fig. 3 a as ‘REM-’, one notes the change of the shift field sign while the H_EB_ magnitude (absolute value) depends on the spacer thickness exactly the same like after ‘REM+’ cooling. Comparison of these two ‘REM+’ and ‘REM-’ H_EB_ dependencies clearly indicates that it is a magnetic state of the ferromagnetic Fe sublayer at RT that defines the direction (+ /− 180°) of interfacial AFM CoO spins and so unidirectional MA of the system below T_N_. This conclusion is moreover valid up to the highest investigated thickness of Au spacer (15Å) for which EB is detectable. This means that the magnetic state of Fe sublayer above CoO Néel temperature can be used to remotely control the spin orientation at the bottom interface of AFM. Other way around, once the particular sense of direction is grafted at the uncompensated CoO(111) interface, it then indirectly biases the Fe magnetization below T_N_ to either positive or negative external magnetic field values.

In the second scenario (d_Au_ induced SRT for the A → B → C sequence) in Fig. 3b, the sample was first cooled down in one of its remanent states. At 80 K the EB behavior (bottom row in Fig. 3b) is clearly governed by the SRT observed at RT (upper panel of Fig. 3b), i.e. starting from the critical SRT thickness d_Au_ = 5 Å the H_EB_ drops down to zero value because the unidirectional MA easy axis switches to Fe[001] in-plane orientation and thus becomes orthogonal to external magnetic field applied during magnetization reversal. In this way the 90° rotation of unidirectional MA axis is realized and presented in Fig. 3b (grey plot). When comparing B and C ROIs one can notice unusual evolution of magnetic hysteresis loops. These two regions are equally distant from the white line marking the SRT border in the two-dimensional (d_Fe_, d_Au_) space presented in Fig. 2a, which normally would result in exactly the same anisotropy field values determined from hard-axis [1 $\overline{1 }$ 0] magnetic hysteresis loops. Clearly, this is not the case for the red loops presented in Fig. 2 b (upper panel) as the system saturates at ~ 450 Oe and at ~ 300 Oe for the B and C ROIs, respectively. This seeming discrepancy can be precisely explained having in mind that freezing of interfacial CoO spins and easy axis of unidirectional MA are determined by the Fe magnetization orientation above Néel temperature of CoO. At 300 K the Fe magnetization in B ROI is oriented along Fe[001] direction and for this reason also the low temperature EB acts as additional (unidirectional) MA that promotes [001] orientation and thus make the loop in **H ||** [1$\overline{1 }$0] MOKE geometry harder. Contrary, for the C ROI (‘chimney’) the Au spacer is thick enough to fully suppress the EB interaction and consequently the corresponding **H ||** [1$\overline{1 }$0] anisotropy field is strongly reduced as compared to B ROI. The same effect can be observed for the E and F ROIs in Fig. 2b but in this case hard-axis magnetic hysteresis loops in **H ||** [001] geometry (grey) should be analyzed. Also please note that the low temperature critical SRT thickness remains unchanged as the Au thickness increases from B, E (significant EB) to C, F (H_EB_ = 0) regions. This is manifested by almost perfectly flat white border line in Fig. 2 a for d_Au_ > 8 Å). This means that although EB strongly contributes to the effective MA of Fe it surprisingly does not shift the critical SRT thickness, which at first glance may look like contradiction. This can be also explained by the fact that for E → B path the 80 K H_EB_ rapidly jumps to zero value exactly at the sample region where the SRT is observed at 300 K. In other words, in such zero-field cooled (ZFC) exchange biased system, the effective MA (including its unidirectional contribution) of Fe continuously decreases and reaches non-zero value (still unidirectional MA contributes) at the critical SRT region. For example in case of E → B path this can be observed close to d_Fe_ ~ 70 Å. Further increasing of Fe thickness leads to sudden one-jump drop and change of sign of the effective MA value as its last (unidirectional) component switches to orthogonal in-plane direction. The last effect is triggered by the particular MA landscape above CoO Néel temperature.

The situation changes significantly when the field cooling procedure (FC) is applied, i.e. the magnetization of Fe is aligned by the external magnetic field along [1$\overline{1 }$0] in-plane direction for all Au thicknesses followed (SRT is suppressed by the external magnetic field) as the system passes CoO Néel temperature. In these circumstances the low temperature EB no longer exhibits a jump of the shift field to its zero value and instead, above Au single monolayer it continuously decays with increasing spacer thickness, similarly to what was observed in Fig. 3a (no SRT) scenario. Note however, that for d_Au_ above ~ 5 Å Fe magnetization is oriented along Fe[1$\overline{1 }$0] only when forced by external magnetic field, for example during FC cooling. Once the field is released (H is set to zero value after FC procedure) at 80 K the Fe magnetization switches to its intrinsic Fe[001] easy axis while interfacial CoO spins remain frozen along Fe[1$\overline{1 }$0] direction. That is why in the whole Au thickness range H_EB_ is large even though magnetic hysteresis loops change from almost square, typical for easy axis for d_Au_ < 5 Å to hard-like (but still exchange biased) for thicker Au spacer. Such two exemplary loops are shown in Fig. 2S in Supplementary Material. Described above MOKE measurements obviously provide direct information about unidirectional MA axis and EB but can also be treated as an indirect probe of the orientation of interfacial antiferromagnetic spins in CoO. The inset in Fig. 3b provides direct confirmation of these conclusions, namely the 80 K results obtained using X-ray Magnetic Linear Dichroism (XMLD)^46,47^ measurements at the PIRX beamline^48^ of National Synchrotron Radiation Centre Solaris in Kraków^49^. In case of AFM CoO, the XMLD magnitude is routinely defined by the so called R_L3_ ratio of the two selected out of four peaks of intensity around L3 absorption edge of Co in X-ray absorption spectra^7^, please see Fig. 3S in Supplementary Material for the R_L3_ ratio definition. The R_L3_(d_Au_) dependence is plotted in the inset of Fig. 3b for both ZFC and FC cooled sample. A change of the R_L3_ between d_Au_ = 0 and 2.5 Å corelates well with the observed enhancement of H_EB_ at this thickness of Au, which in the most simplified picture can be interpreted as the increased population of frozen interfacial CoO spins oriented along Fe[1–10] in-plane direction. Furtherly, for the ZFC cooled sample, around critical Au thickness ~ 5 Å the XMLD magnitude (R_L3_) decreases even more which again agrees well with the H_EB_ disappearance and room temperature SRT in Fe. Again the situation changes after the FC procedure was applied in the PIRX measurement chamber, specifically the R_L3_ ratio, instead of rapid one-step jump at SRT, continuously decreases its value as the interaction mediated by Au becomes weaker. Finally, at the ‘chimney’ position the XMLD is almost identical for both ZFC and FC cooled sample, as expected when FM-AFM interaction is no longer present.

In the third scenario (Fig. 3c), at RT the Fe thickness induced SRT is observed at d_Fe_ ~ 70 Å and consequently after the ZFC cooling a corresponding sudden drop of H_EB_ to zero value takes place again. Such reduction of the shift field at the SRT critical thickness is no longer observed after the FC procedure with external magnetic field applied either along Fe[1$\overline{1 }$0] or antiparallel Fe[$\overline{1 }$10] in-plane direction, leading to negative or positive values of the EB shift field, respectively. These results are marked in Fig. 3c as ‘FC + ‘ and ‘FC- ‘, respectively, in analogy to plots ‘REM + ’ and ‘REM-’ in Fig. 3a. (Note that applying the ‘FC + ’ or ‘FC- ‘ procedure in the first of the analyzed scenarios (Fig. 3a) leads to exactly the same results as cooling in ‘REM + ’ and ‘REM-’ states as no SRT takes place for this sample region.)

**Figure 4:**
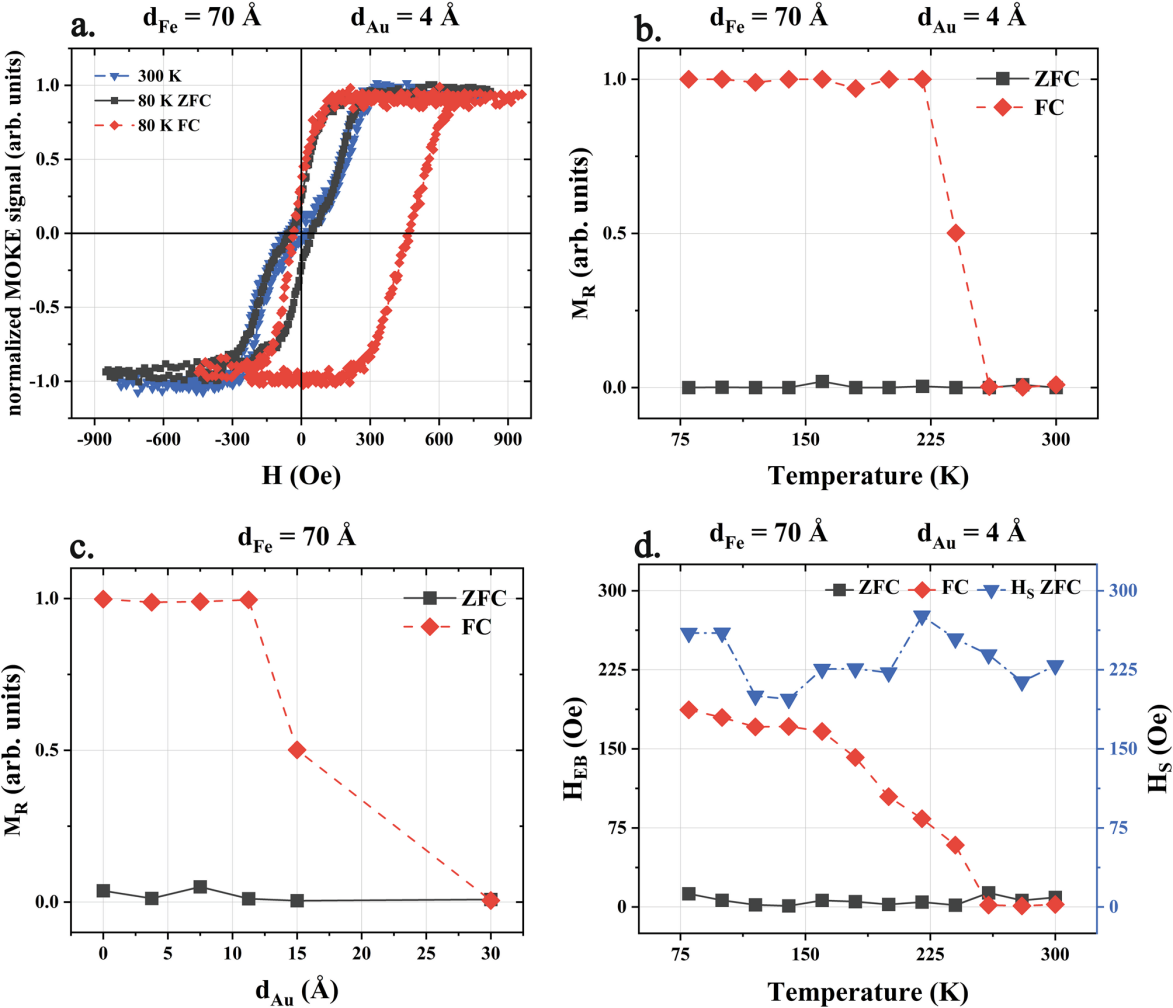
(**a**) Three exemplary magnetic hysteresis loops measured in **H** || [001] MOKE geometry for sample region corresponding to d_Fe_ = 70 Å, d_Au_ = 4 Å. (**b**) 80 K magnetization in remanent state M_R_(d_Au_) dependencies for both FC and ZFC cooled sample. (**c**) Magnetization in remanent state M_R_(T) temperature dependencies acquired after FC (red) and ZFC (grey) cooled sample. Corresponding EB shift field temperature dependence is shown in (**d**) where also the anisotropy field H_s_ (blue) is plotted for the ZFC case.

In the previous paragraphs we showed the interplay between FM and AFM sublayers and these results can be summarized as follows. Room temperature orientation of Fe magnetization freezes the particular orientation of interfacial AFM spins as the system passes its Néel temperature and as a result, at low temperature these frozen AFM spins induce EB and unidirectional MA in Fe, however the easy axis of uniaxial MA of Fe remains unchanged. In the following part of this report we will provide examples of how interaction with AFM can also reorient the easy axis of effective magnetic anisotropy of FM layer. In order to do this one has to focus on sample region where possibly large EB can be induced and therefore the most natural choice is rather thin Fe and thin Au spacer, such results are shown in Fig. 4.

In Fig. 4a three magnetic hysteresis loops are shown as measured in **H ||** [001] MOKE geometry for the selected sample region close to ROI “D", specifically corresponding to d_Fe_ = 70 Å and d_Au_ = 4 Å thicknesses. First of these loops (blue in Fig. 4a) was measured at 300 K and so it is a typical ‘hard-like’ and exchange bias-free magnetic hysteresis curve. After ZFC cooling of the sample to 80 K, the magnetic hysteresis loop (grey in Fig. 4a) changes a little however it still remains ‘hard-like’, with H_EB_ = 0 and importantly, its anisotropy field (H_s_) is almost unchanged as compared to 300 K loop. The last observation results from the very weak temperature dependence of effective MA of Fe on W(110). Interestingly, after FC cooling of the sample in external magnetic field **H ||** [001], not only the non-zero H_EB_ is induced but also the low temperature magnetic hysteresis loop (red in Fig. 4a) becomes more square-like and characteristic for magnetization reversal along easy axis. This can be explained by the large contribution of additional (unidirectional) MA which in this case favors the [001] orientation of Fe magnetization. Such effect can be also documented in much wider range of the Au thickness, please see Fig. 4b where 80 K magnetization in remanent state (M_R_) is plotted as a function of d_Au_ for ZFC and FC cooled sample. Clearly, below ~ 15 Å thick Au spacer the EB is a strong enough [001] MA source which overcomes the intrinsic [1$\overline{1 }$0]-like MA of Fe. At the ‘chimney’ area the Fe-CoO interaction is fully suppressed and so even after FC procedure the Fe magnetization switches again to Fe[1$\overline{1 }$0] direction. Similarly, for a chosen sample ROI, the Au mediated Fe-CoO exchange interaction can be also continuously decreased by increasing temperature. In Fig. 4. c and d the magnetization in remanent state and EB shift field dependencies on the temperature are shown, respectively. Obviously, for the ZFC cooled sample both M_R_ and H_EB_ values are almost zero within the whole studied temperature range. After the FC procedure the M_R_ becomes equal to 1 and H_EB_ ~ 180 Oe is induced at 80 K, the latter continuously decreases with increasing temperature. In this way also the [001] contribution to MA of the Fe decreases and at some critical temperature (~ 260 K) a temperature induced SRT takes place and Fe magnetization switches to its intrinsic [1$\overline{1 }$0] easy axis, note the M_R_ jump to zero value in Fig. 4c. Such temperature induced SRT can be induced only in strongly exchange biased system as it originates fully from the temperature dependence of EB, whereas the effective MA of EB-free system (after zero field cooling) is almost independent on the temperature as proved by the H_s_(T) plot in Fig. 4d (blue).

In conclusion, specific choice of the ROI in the two-dimensional (d_Fe_, d_Au_) space allows to precisely tune uniaxial and unidirectional magnetic anisotropy of Fe as well as the strength of Fe-CoO indirect, Au-mediated exchange interaction. As a result, a variety of magnetic moments orientations in both ferromagnetic and antiferromagnetic sublayers can be stabilized and reorientations between particular magnetic configurations can also be triggered either by changing the Fe or Au thickness or temperature. The choice of the magnetic state of the ferromagnetic layer above the Néel temperature of the antiferromagnet along with applied cooling procedure is a decisive factor for low temperature orientation of interfacial AFM spins.

### Supplementary Information

Supplementary Information.

## Data Availability

The datasets used and/or analyzed during the current study available from the corresponding author on reasonable request.
