# Supplementary material for: Tunable interplay between exchange coupling and uniaxial magnetic anisotropy in epitaxial CoO/Au/Fe trilayers
Source: Sci Rep. 2023 Jul 5;13:10902. doi: 10.1038/s41598-023-38098-6 (PMC10322835; doi:10.1038/s41598-023-38098-6)
Supplement: Supplementary file 1 — Supplementary Information. [file 41598_2023_38098_MOESM1_ESM.docx]

Supplementary material for:

**Tunable interplay between exchange coupling and uniaxial magnetic anisotropy in epitaxial CoO/Au/Fe trilayers.**

H. Nayyef^a^, E. Świerkosz^a^, W. Janus^a^, A. Klimeczek^a^, M. Szpytma^a^, M. Zając^b^, P. Dróżdż^c^, A. Kozioł-Rachwał^a^, T. Ślęzak^a^, M. Ślęzak^a*^

*^a^* AGH University of Krakow, Faculty of Physics and Applied Computer Science, Kraków, Poland

*^b^* National Synchrotron Radiation Centre SOLARIS, Jagiellonian University, Kraków, Poland
*^c^*  Institute of Physics, Maria Curie-Sklodowska University, Lublin, Poland

*mislezak@agh.edu.pl

In Fig.1S of this supplementary material, we show the LEED diffraction pattern which were captured while preparing our CoO(111)/Au(111)/Fe(110) system. The dark spots in the LEED snapshots refer to the diffraction pattern of the surface of the system at each preparation step. Accordingly, ball models of Fe(110), Au(111) and CoO(111) are also presented in the lower panel of Fig. 1S.


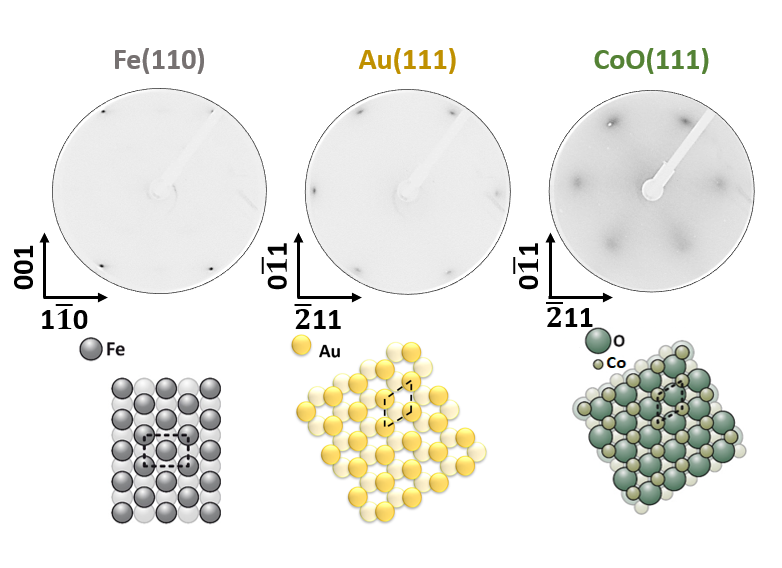


Fig. 1S (Supplementary) Diffraction LEED patterns of the surface of: Fe(110), electron energy E = 50 eV, Au(111)/Fe(110), E = 55 eV and CoO(111)/Au(111)/Fe(110) E = 80 eV. In the bottom panel corresponding ball models are presented.

Below, in Fig. 2S we present two exemplary magnetic hysteresis loops for the scenario analyzed in Fig. 3b of the article main body (d_Au_ induced SRT for the A🡪B🡪C sequence). When the field cooling procedure is applied, i.e. the magnetization of Fe is aligned by the external magnetic field along [1$\bar{1}$0] in-plane direction for all Au thicknesses followed (SRT is suppressed by the external magnetic field) as the system passes CoO Néel temperature. In the whole Au thickness range EB is large even though magnetic hysteresis loops change from almost square, typical for easy axis for d_Au_ < 5 Å to hard-like (but still exchange biased) for thicker Au spacer.


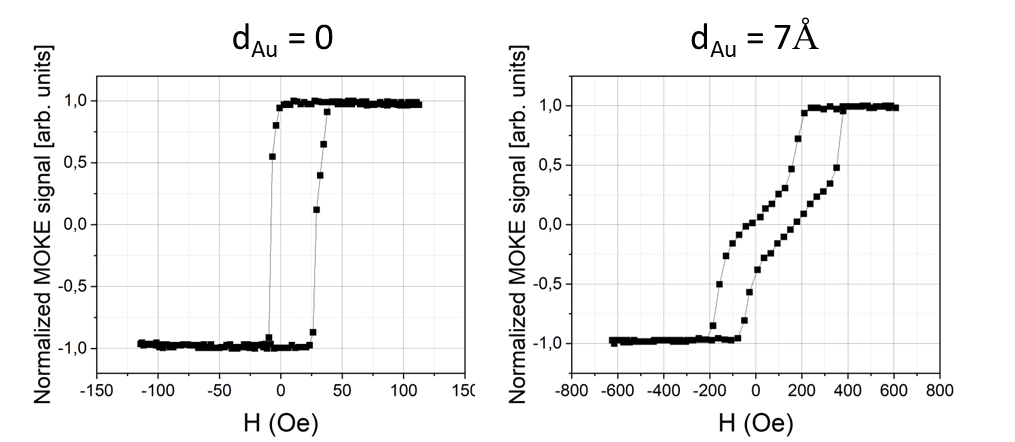


Fig. 2S. (Supplementary) Two exemplary magnetic hysteresis loops measured after FC cooling the sample to 80 K. See details in the description of Fig. 3b in the main text.

In Fig.3Sa the geometry of the XMLD measurements performed at the PIRX beamline in Solaris facility in Kraków is shown. The data presented in the inset of Fig.3b of the article’s body was obtained in a normal incidence geometry (φ = 0) and incoming X-rays linear polarization parallel to the Fe[1-10] direction (**E** || Fe[1-10]). XAS measurements were performed in the Total Electron Yield (TEY) mode. Fig.3Sb of this supplementary material shows an exemplary XAS spectrum acquired around L3 edge of Co. We can define the so called R_L3_ ratio as R_L3_ = P1/P2 (the ratio of the first and the fourth peaks), i.e. it is defined as the ratio of the XAS intensity at 777.2 eV and at 779.8 eV energies.


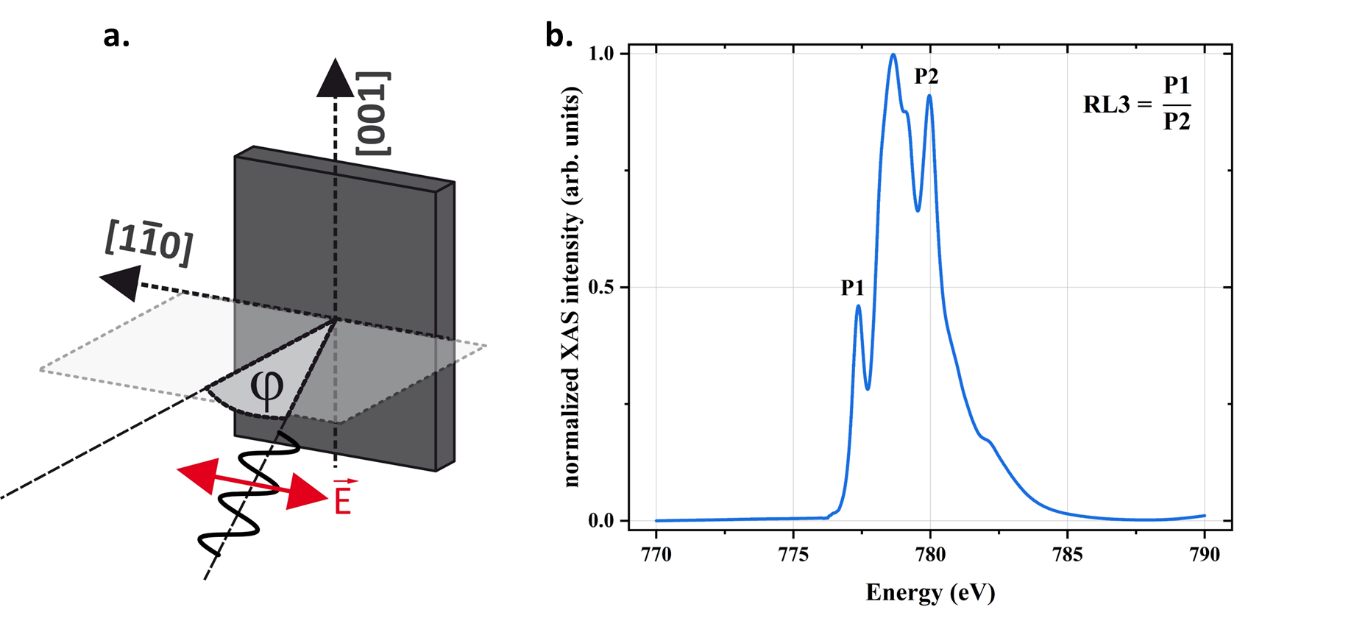


Fig.3S (Supplementary) (a) Geometry of the XMLD measurements performed at the PIRX beamline in Solaris facility (b) Exemplary XAS spectrum captured around L3 edge of Co together with definitions of P1 and P2 intensities used for the R_L3_ determination.

Below we also present exemplary XMLD (Fig. 4S) and XMCD (Fig. 5S) spectra. All XAS measurements were performed at the PIRX beamline in Solaris synchrotron. The beam spot size (FWHM) on the sample was 40 µm x 200 µm (vertical x horizontal). This corresponds to the averaging of magnetic properties over a finite thickness intervals ∆d_Fe_ = ~ 1 Å and ∆d_Au_ = ~ 0.8 Å for the Fe and Au wedges, respectively.


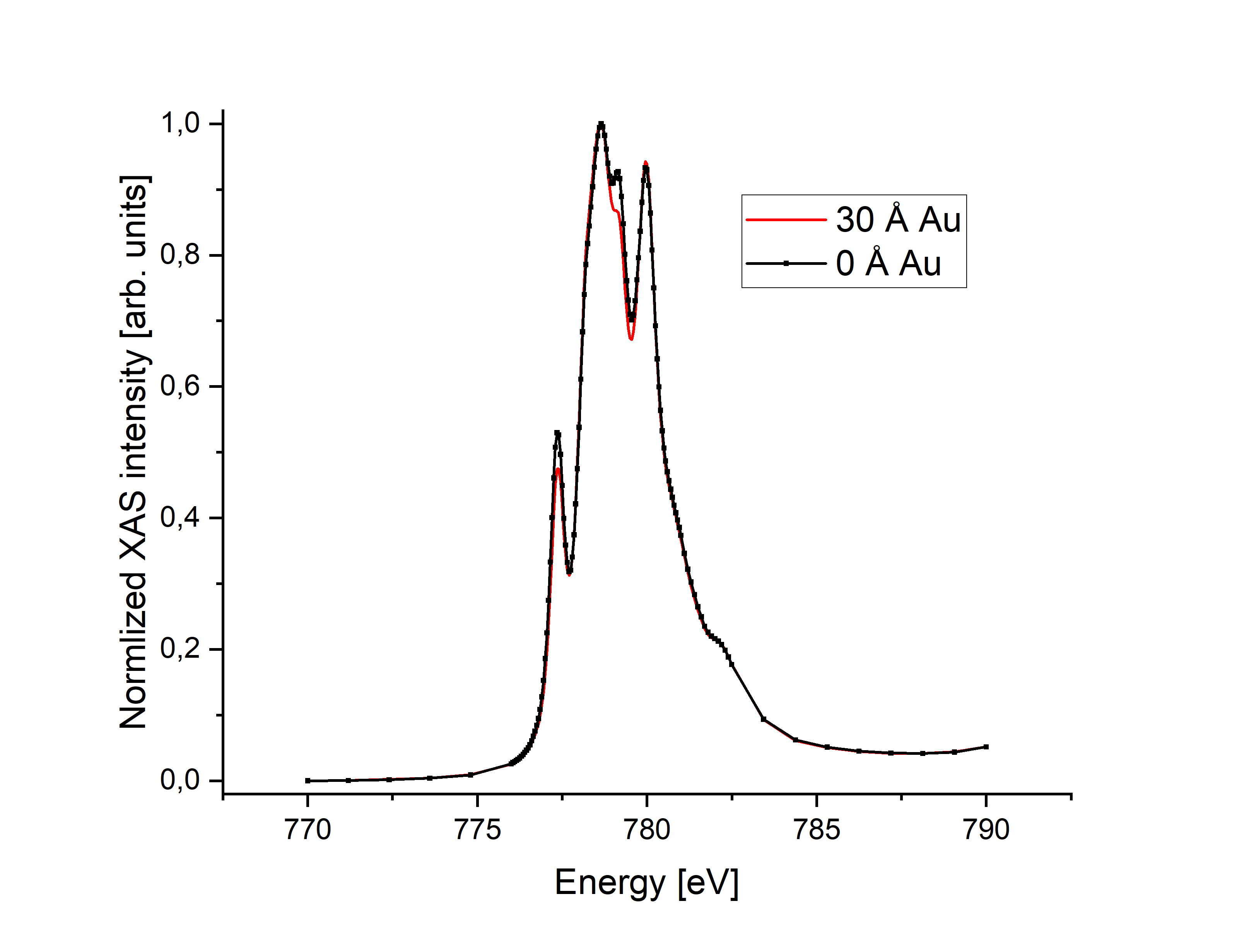


Fig.4S. Two exemplary 80 K XMLD spectra for zero field cooled (ZFC) sample chosen from the series of measurements used for the inset in Fig.3b. From these spectra R_L3_ ratio presented in the inset of Fig.3b in the main article was determined.


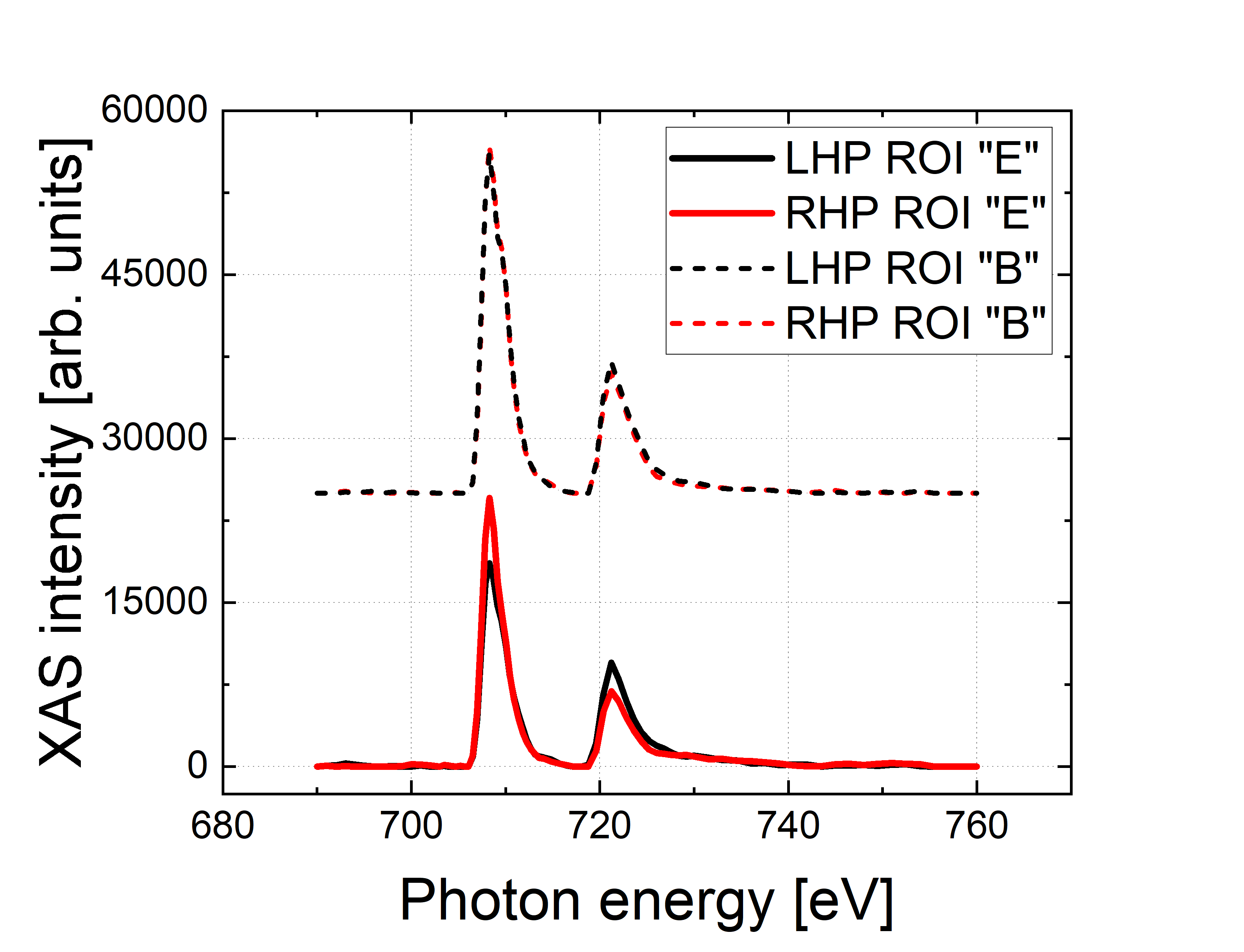


Fig.5S. Two exemplary XMCD spectra collected for ROIs “E” (solid lines) and “B” (dashed line) so before (large XMCD) and after (no XMCD) Fe thickness induced SRT, respectively. LHP and RHP stand for the left- and right- handed circular polarization of incoming X-rays. The spectra for ROI “B” were intentionally shifted along vertical axis to allow their presentation with ROI “E” data in one plot.

In Fig. 6S we present XAS spectra around L3 absorption edge of Fe for CoO/Fe bilayer (no Au spacer) and for ~1 monolayer (ML) of Au in CoO/1 ML Au/Fe trilayer. One can note, that in case of no Au spacer spectrum slightly deviates from typical metallic Fe spectra, indicating partial oxidation of the topmost Fe layers which results from reactive deposition of CoO overlayers. Independently on the protective role of Au spacer, 2 Å – thick metallic Co was grown on the whole sample area before the reactive deposition of CoO in order to minimize the oxidation of Fe.


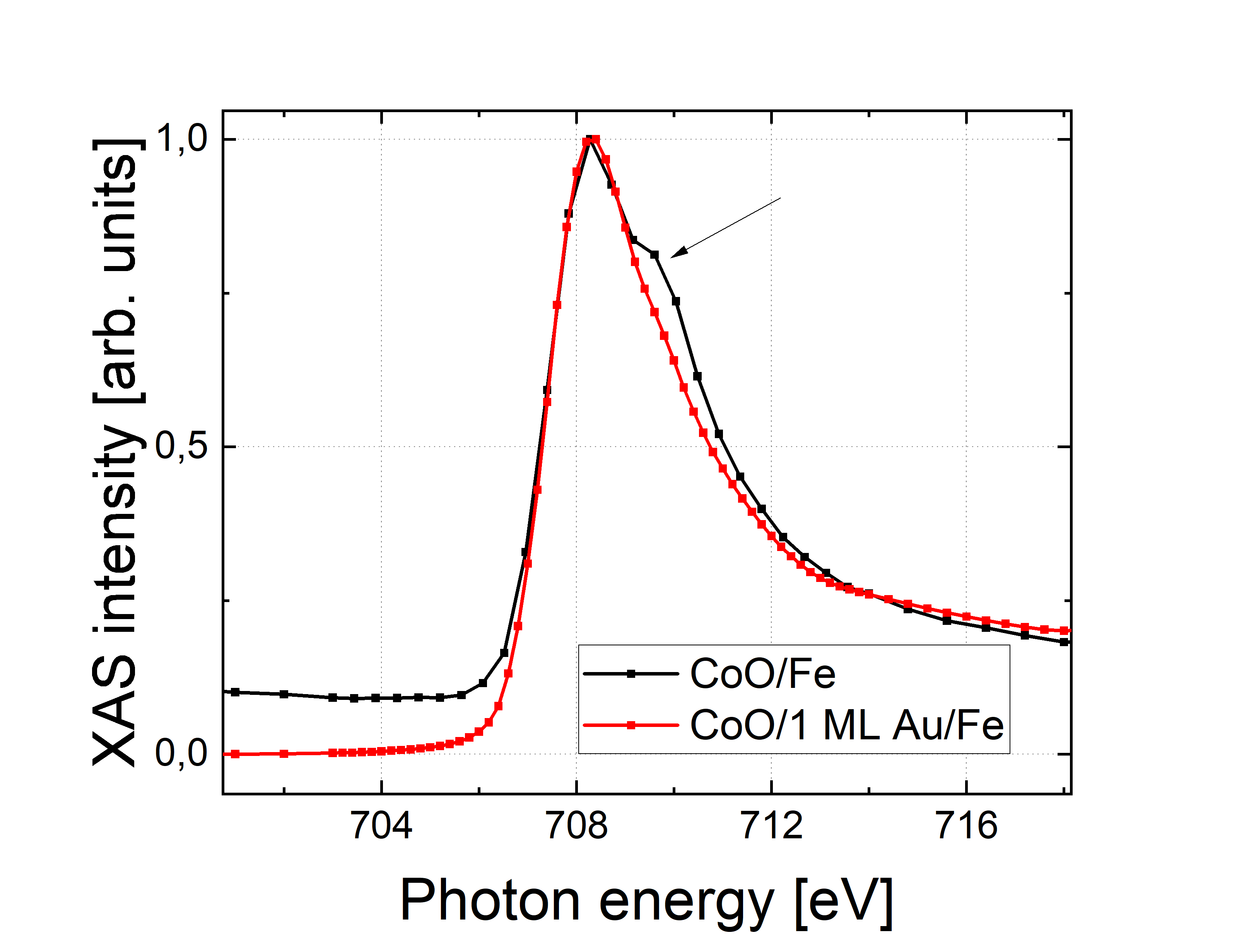


Fig. 6S. XAS spectra around L3 absorption edge of Fe for CoO/Fe bilayer (no Au spacer) and for ~1 monolayer (ML) of Au in CoO/1 ML Au/Fe trilayer. Arrow marks the change of intensity profile resulting from the slight oxidation of uncovered Fe surface during initial stage of the CoO growth.
